# Multisensory Oddity Detection as Bayesian Inference

**DOI:** 10.1371/journal.pone.0004205

**Published:** 2009-01-15

**Authors:** Timothy Hospedales, Sethu Vijayakumar

**Affiliations:** Institute of Perception, Action and Behaviour, School of Informatics, University of Edinburgh, Edinburgh, United Kingdom; Duke Unviersity, United States of America

## Abstract

A key goal for the perceptual system is to optimally combine information from all the senses that may be available in order to develop the most accurate and unified picture possible of the outside world. The contemporary theoretical framework of ideal observer maximum likelihood integration (MLI) has been highly successful in modelling how the human brain combines information from a variety of different sensory modalities. However, in various recent experiments involving multisensory stimuli of uncertain correspondence, MLI breaks down as a successful model of sensory combination. Within the paradigm of direct stimulus estimation, perceptual models which use Bayesian inference to resolve correspondence have recently been shown to generalize successfully to these cases where MLI fails. This approach has been known variously as model inference, causal inference or structure inference. In this paper, we examine causal uncertainty in another important class of multi-sensory perception paradigm – that of oddity detection and demonstrate how a Bayesian ideal observer also treats oddity detection as a structure inference problem. We validate this approach by showing that it provides an intuitive and quantitative explanation of an important pair of multi-sensory oddity detection experiments – involving cues across and within modalities – for which MLI previously failed dramatically, allowing a novel unifying treatment of within and cross modal multisensory perception. Our successful application of structure inference models to the new ‘oddity detection’ paradigm, and the resultant unified explanation of across and within modality cases provide further evidence to suggest that structure inference may be a commonly evolved principle for combining perceptual information in the brain.

## Introduction

Bayesian ideal observer modelling is an elegant and successful approach to understanding human perception [1]. One particular domain in which it has seen much success recently is that of understanding multisensory integration in human perception [2]. In this context, the ideal observer essentially specifies how the information from each sense should be optimally *weighted* in creating the unified percept of a particular source observed with multiple cues or modalities. As an intuitive example, consider that when walking your dog in the park on a clear day, you automatically and easily locate it visually, without relying much on the auditory localization of it's bark – because the optimal visual weight in this case is much larger. This has proven a good qualitative explanation of numerous experiments including audio-visual [3], [4], visual-haptic [5], [6], texture-stereo [7], [8] and texture-motion [9] pairs among others. The near optimal sensor fusion observed widely across these different pairs of senses suggests that this may be a common principle of sensory integration in humans.

However, these models have broken down when, in addition to uncertain noisy stimuli, the observer is uncertain about the *correspondence* of the multisensory observations, i.e., when it is not clear whether two observations were indeed caused by the same source of interest or not. Consider another intuitive example. When your dog has run off while walking in the forest, it may not be clear whether you should search for it: (i) in the direction it ran off in (prior information), (ii) in the direction you see moving leaves (visual information), (iii) in the direction you hear a bark from (auditory information) or (iv) some particular weighted combination of (i)–(iii). If you hear a bark from the same direction as you last saw the dog, and see moving leaves at a completely different location, you might assume you heard your dog's bark – discounting the moving leaves entirely as being due to another animal – and search in a direction somewhere between where it was last seen and the bark. Alternately, if the leaves move where you last saw your dog, but the bark comes from elsewhere, you might do the opposite – discounting the bark as some other animal instead. Unlike maximum likelihood integration (MLI), the Bayesian structure inference approach provides a systematic and quantitative solution to these kinds of problems.

In cases like the example described, MLI models have failed to explain the experimental data. Fundamentally, this is because although MLI models are derived from a probabilistic perspective, they are not Bayesian about the uncertain *correspondence* between the observations. Hence, we also refer to them as *mandatory fusion* models because they assume observations correspond. This is in contrast to *structure inference* (or *causal inference*) models which also infer the causal structure of the multisensory observations.

Very recent work has begun to apply a complete Bayesian structure inference perspective [10], [11] to experiments with such uncertainty [12], [13], [14], and have provided a good explanation for the perceptual process in these cases [15], [16]. However, to date, all existing work on models of structure inference in human perception has been applied to paradigms involving *direct estimation* of the stimulus. In this paper, we consider a related paradigm in multi-sensory perception – that of *multisensory oddity detection*. We show how multisensory oddity detection is a novel and interestingly unique paradigm that require careful considerations during modeling, how structure inference of causal uncertainty applies in this context, and how it can explain and unify a pair of experiments ([17]) where MLI previously failed dramatically.

In the remainder of this section, we review standard MLI ideal observer modelling for sensor fusion, and show – by way of theoretical argument as well as a concrete experimental example – why the naive application of mandatory fusion MLI approaches qualitatively fail to explain human multisensory oddity detection.

### Standard Ideal Observer Modelling for Sensor Fusion

In the Bayesian modelling approach to perception, a generative probabilistic model for the perceptual process is defined. This describes the way in which signals are generated by a source, and how they are then observed - including any distorting noise processes. Predictions made by the results of optimal inference in this model can then be compared to experimental results.

**Figure 1:**
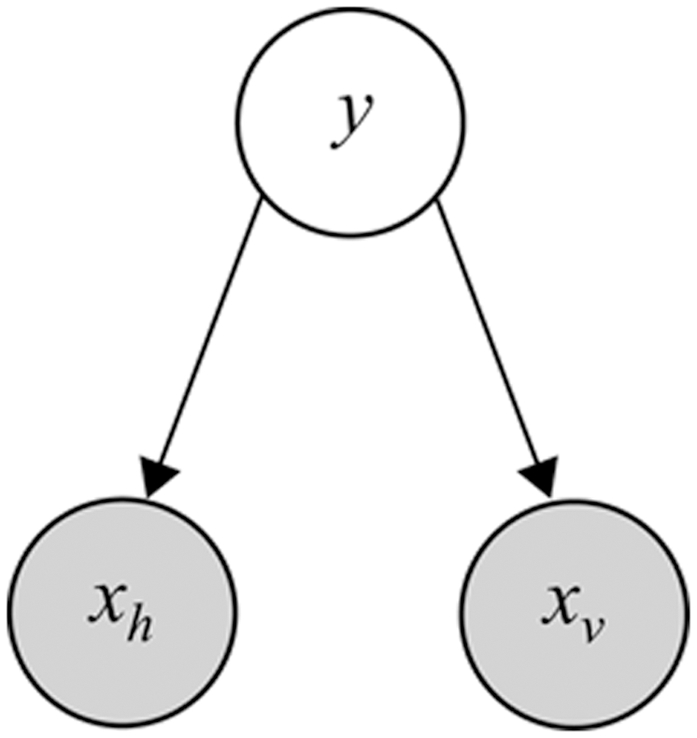
Standard sensor fusion model. Bar size *y* is inferred on the basis of haptic and visual observations 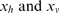 [17].

Standard sensor fusion theory assumes that multisensory observations 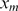 in modalities *m* are generated from some source *y* in the world, subject to independent noise in the environment and physical sensor apparatus, e.g., 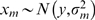. The sensors may have different variances 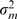. For example, in [5], subjects make haptic 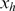 and visual 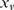 observations of a bar's height *y*, and must report their best combined estimate 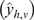 of the true height. This is an inference problem which can be represented by the generative graphical model shown in Fig. 1. Under this particular noise model, the posterior distribution of the height estimate is a Gaussian 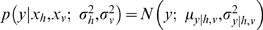, with mean and variance given by eqs. (1–2):
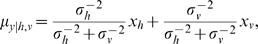
(1)

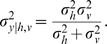
(2)For this Gaussian posterior 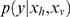, the optimal estimate to make 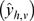 under the standard mean square cost function [18] is the mean of the posterior, which turns out to be the precision (inverse variance) weighted average of the individual observations (eq. (1)).

Psychophysics experiments such as [2], [5] typically test multisensory perception for optimality by matching to the ideal observer performance in two ways. Firstly, the variance of the optimal response 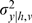 is less than the variance of the individual observations 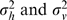 (eq. (2)). Therefore, the distribution of a human's responses 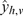 to a multisensory stimulus should have a lower variance than their responses 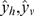 to the uni-modal stimuli. Secondly, the multisensory response of the ideal observer is the precision weighted mean of the uni-modal observations (eq. (1)). Therefore, experimentally manipulating the variances 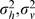 of the individual modalities should produce the appropriate changes in the human perceptual response 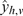. These quantities can be determined directly in direct estimation experiments (e.g., [12], [13]) or indirectly via fitting a psychometric function in 2-alternative forced choice experiments (e.g., [2], [5]).

### Oddity Detection

In the direct estimation scenarios, subjects try to make a *continuous estimate* of a particular unknown quantity *y*, such as height of the bar or spatial stimulus location based on noisy observations 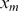, such as visual and haptic heights or auditory and visual locations, respectively. In contrast, in the *oddity detection paradigm*, subjects observe 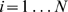 separate stimuli 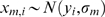 and must make a discrete estimation 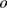 of the “odd” stimulus 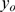 from amongst the 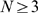 options 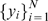. Depending on the experimental paradigm, the odd stimulus may be detectable because it is, for example, larger or smaller than the other stimuli.

*Multisensory* oddity detection is a particularly interesting problem to study for various reasons. Notably, it provides novel paradigms for manipulating the oddity. Specifically, a particular stimulus might be the same as the others when averaged over its modalities of perception (as required by mandatory fusion MLI), while each individual stimulus modality could simultaneously be radically discrepant. Such stimuli would be known as *perceptual metamers*, meaning that although they would be physically distinct, they would be perceptually indistinguishable under this theory of cue combination. This provides a new and interesting test of Bayesian perception, because if the nervous system was to necessarily fuse the modalities first and use the fused estimates to detect oddity, then it would not be able to detect such metamers. If on the other hand, the nervous system made an inference about the structure of the observations, it could detect such stimuli on the basis of structure (correspondence) oddity. In the following section, we formalize this inference paradigm and look in detail at a pair of experiments that tested oddity detection and found MLI mandatory fusion models unsatisfactory in explaining the data completely.

### Human Multisensory Oddity Detection Performance

Hillis et al. [17] studied multisensory oddity detection in humans using *N* = 3 options in two conditions: visual-haptic cues for size (across-modal cues) and texture-disparity cues for slant (within-modal cues). For ease of comparison, we describe this experiment in some detail, and will formalize the oddity detection problem and our solution to it in the context of this experiment. It should be noted that our approach can trivially be generalized to other conditions, such as more modalities of observation and selecting amongst 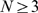 options.

**Figure 2:**
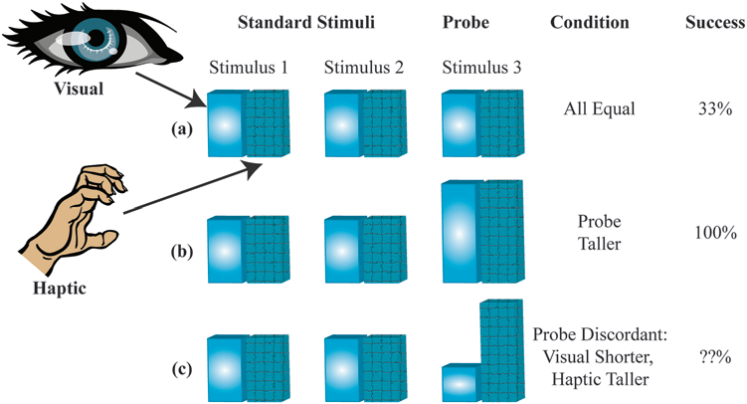
Schematic of visual-haptic height oddity detection experimental task from [17]. Subjects must choose the odd probe stimulus based on haptic (textured bars) and visual (plain bars) observation modalities. a) Probe stimulus is the same as the standard stimuli: detection at chance level. b) Probe stimulus bigger than standard: detection is reliable. c) Haptic and visual probe modalities are discordant: detection rate will depend on cue combination strategy.

Three stimuli are presented in two modalities *v* and *h* (Fig. 2). (To lighten the discussion, we will refer generally to the visual-haptic (*v*-*h*) modalities when discussing concepts which apply to both the visual-haptic and texture-disparity experiments.) Two of the stimuli are instances of a fixed standard stimulus 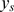 and one is an instance of the (potentially odd) probe stimulus 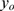. The standard stimuli are always *concordant*, meaning that there is no experimental manipulation across modalities; so 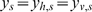. The probe stimulus 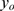 is experimentally manipulated across a wide range of values so that the visual and haptic sources, 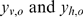, may or may not be similar to each other or to the standard 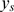. The subject's task is to detect which of the three stimuli is the probe. If all the stimuli are concordant and the probe is set the same as the standard 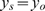, then we expect no better than random (33%) success rate (Fig. 2a). If all the stimuli are concordant and the probe discrepancy is set very high compared to the standard, then we expect close to 100% success rate (Fig. 2b). However, if the probe stimulus is experimentally manipulated to be *discordant* so that 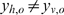, then the success rate expected will depend on precisely how the subjects combine their observations of 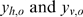 (Fig. 2c). The two dimensional distribution of detection success/error rate as a function of controlled probe values 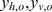 can be measured and used to test different theories of cue combination.

For a single modality, e.g. *h*, the error rate distribution for detection of the probe 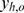 can be modelled as a one dimensional Gaussian bump centered around the standard 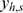. (If 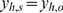 then detection of the odd stimulus will be at chance level, if 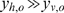 then detection of the odd stimulus will be reliable, etc.) The shape of the two dimensional performance surface for multi-modal probe stimulus detection 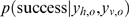 can be modelled as a two dimensional bump centered at 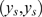. Performance *thresholds* (the equipotentials where 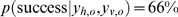) are computed from the performance surfaces predicted by theory and those of the experimental data. The cue combination theories are evaluated by the match of their predicted thresholds to the empirical thresholds.

### Basic Cue Combination Theories

**Figure 3:**
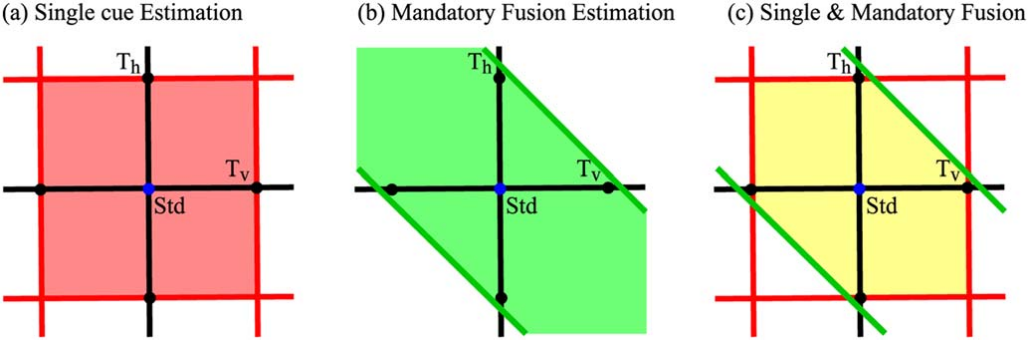
Oddity detection predictions of the naive cue combination models. (a) Detection based on individual cues only. (b) Detection based on a single fused estimate 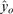. (c) Detection based on both individual cues and a single fused estimate. Shaded areas indicate regions below threshold probability of correct detection. The standard stimulus 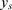 is indicated by a blue dot in the centre of each plot. 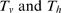 indicate uni-modal visual and haptic thresholds respectively. Coloured lines indicate multi-modal detection rate contours.

To parameterize models for testing, the observation precisions first need to be determined. Following standard practice for MLI modelling, Hillis et al. [17] measure the variances of the uni-modal error distributions and then, use these to predict the multi-modal error distribution under mandatory fusion cue combination theory (refer eqs. (1) and (2)). (In the Results section, we will discuss why this naive approach is not quite correct for this experiment.) On this basis, Hillis et al. identify a set of four basic theories (Fig. 3) for how the brain might perform the multisensory oddity detection task, each with distinct predictions about the nature of the threshold of probe detection around the standard stimulus (Fig. 3, blue dot):

1. The probe stimulus might be detected based on one observation modality *i* only, ignoring the other entirely. This predicts a band, of width determined by the uni-modal variance 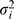, within which the probe is too similar to the standard to be reliably detected. The band would be perpendicular to the axis of cue *i* and centered around the standard stimulus 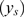 (Fig. 3a, red lines).
2. The probe stimulus might be detected based on one cue and then the other, in a cascaded sequence. This predicts a rectangle about the standard 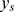 within which the probe is too similar to the standard to be reliably detected. The dimensions of the rectangle are given by the intersection of the two bands from the first option (Fig. 3a, red square).
3. It might compute a single fused estimate 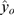 based on the two observations 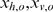 (eqs. (1) and (2)) and then, discriminate purely based on this estimate. In this case, although both cues are now being used, some combinations of cues would produce a metameric probe, i.e., physically distinct but perceptually indistinguishable. Specifically, if we parameterise the probe stimuli as 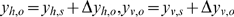, then along the line where 
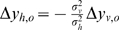
, the fused estimate is the same as the standard 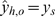 and the probe would be undetectable. The band of non-detection is therefore along the cues-discordant diagonal ((Fig. 3b), green band). The orientation and width of this band are determined by the ratio 
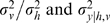
, respectively. Performance along the cues-concordant diagonal is, however, improved compared to the single cue estimation cases (compare quadrants 1 and 3 in Fig. 3a,b) because the combined variance is less than the individual variances 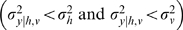. This is the standard mandatory fusion MLI theory.
4. It might perform combined and single cue detection in sequence, giving a prediction which is the intersection of the second and third options (Fig. 3c, yellow area).

### Human Performance

**Figure 4:**
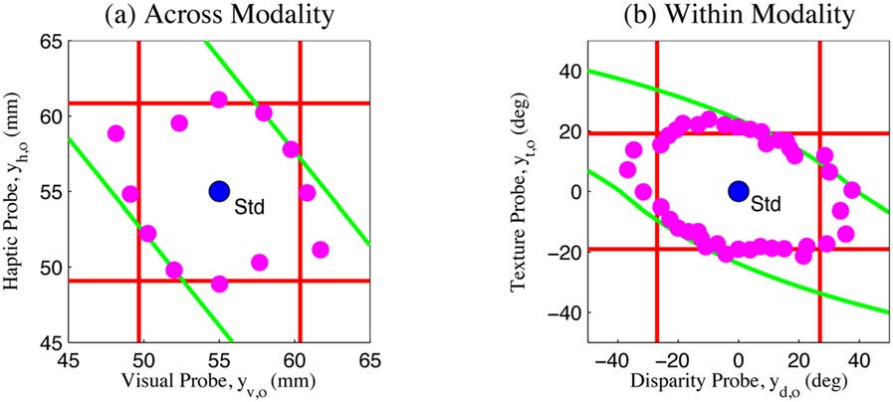
Oddity detection predictions and experimental results. Experimental data for two sample subjects from [17]. (a) Visual-haptic experiment. (b) Texture-disparity experiment. Red lines: Observed uni-modal discrimination thresholds. Green lines: Discrimination threshold predictions assuming mandatory fusion. Magenta points: Discrimination threshold observed experimentally.

Two variants of the experiment were performed, one for size discrimination across visual and haptic modalities (standard: 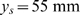), and one for slant discrimination using texture and stereo disparity cues within vision (standard: 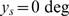). Comparing the threshold predictions (lines) to the results observed by Hillis et al. [17] for two sample subjects (data points) in Fig. 4, there are several points to note: i) In the cues concordant quadrants (1&3), the multi-modal performance is increased compared to the uni-modal performance, as predicted by the fusion theories (magenta points and green lines are inside the red lines in quadrants 1&3). This suggests that some cue combination is taking place, and that the first two basic theories (1, 2) of independent, uni-modal, detection are insufficient. ii) Particularly in the intra-modal case (Fig. 4b), the observed experimental performance is significantly worse in the cues discordant quadrants (2&4) than predicted by any of the basic theories (1, 2, and 4) which allow detection based on individual cues (magenta points are outside of the red lines in Fig. 4(b), quadrants 2&4). In both experiments, the last basic theory (4) of sequential combined and single cue detection also fails, as performance is worse than it predicts (magenta points outside the inner bounding box of lines in Fig. 4, quadrants 2&4).

Since the poor performance in the cues discordant quadrants 2&4 was noted to be less prominent in the inter-modal case (Fig. 4a), Hillis et al. concluded that mandatory fusion applied within (Fig. 4b) but not between (Fig. 4a) the senses [17]. However, even in the intra-modal case, the region of non-detection defined by the magenta points is only extended slightly away from the centre along the cues-discordant diagonal, whereas the mandatory fusion theory predicts that it should extend along an infinite metameric band. The strongest conclusion that can be drawn is therefore that intra-modal perception shows a stronger tendency toward fusion than inter-modal perception.

None of the basic theories proposed (1,2,3,4) explain the qualitative shape of the data well - good performance in the cues concordant quadrants 1&2 as well as a *limited* region of poor performance in the cues discordant quadrants 2&4. In particular, the classical theory of ideal observer maximum likelihood combination which Hillis et al. concluded applied in the within-modal case retains a strong *qualitative* discrepancy with the experimental results (Fig. 4b, green lines and points). In the next sections, we will show how a corrected formalism of the oddity detection problem and structure inference can together explain this data quantitatively and intuitively without the large discrepancy entailed by maximum likelihood, mandatory fusion combination.

### Modelling Oddity Detection

We now introduce the two novel contributions required to model multisensory oddity detection and interpret the results in [17]. Firstly, we will introduce a model selection framework to represent the oddity problem and the explicit inference of the odd stimulus. This is in contrast to the approach of Hillis et al. as described previously, which focused on inference of the latent stimulus and only dealt implicitly with actual identification of the odd stimulus. The explicit representation of oddity is necessary, but as we shall see, it is insufficient to completely understand this multisensory oddity detection problem. We will then introduce the second key step, which is to represent the structure uncertainty in the probe distribution.

### Formalizing Optimal Oddity Detection

Ideal observer theories of cue combination in human multisensory perception have been tested extensively in the form of simple sensor fusion models [2], [5], [6], [9], [19]. Since these experiments are describable by a simple factored Gaussian parametric form (Fig. 1), the optimal computations to use for inference were those described by eqs. (1) and (2).

However, the perceptual task of oddity detection is not actually properly described by the standard factored Gaussian parametric form. This is because the task posed - “*Is stimulus 1, 2 or 3 the odd one out?*” - is actually no longer simply an estimation of a combined stimulus 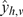. Such an estimation is involved in solving the task, but ultimately the task effectively asks subjects to make a *probabilistic model selection* [20], [21] between three models. (Note that this problem can also be understood as finding the most likely assignment of points in a clustering task. Specifically, consider mixture of Gaussian clustering of three two-dimensional points into two clusters with unknown means.) To understand the model selection interpretation intuitively, consider the following reasoning process: *I have experienced three noisy multisensory observations. I do not know the true values of these three stimuli, but I know they come from two categories, standard and probe. Is it more plausible that: 1. Multisensory stimuli two and three come from one category, and stimulus one comes from another? Or: 2. Stimuli one and three come from one category, and stimulus two comes from a different category? Or: 3. Stimuli one and two come from one category, and stimulus three comes from another?*

With this in mind, to take a Bayesian ideal observer point of view on this experiment, we clearly need a more sophisticated model selection approach than the simple factored sensory fusion approach of Fig. 1. This should *integrate over the distribution of unknown stimulus values* 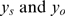 (since subjects are not directly asked about these) in determining the most plausible model (assignment of oddity).

**Figure 5:**
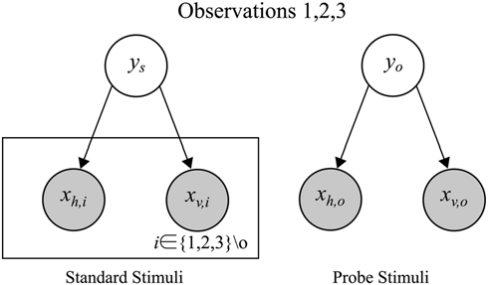
Graphical model for oddity detection by model selection. Three possible models, indexed by *o*, corresponding to each possible assignment of oddity. To compute the stimulus most likely to be odd, compute the evidence for each model 
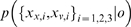
. Standard and probe stimulus values 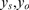 are not directly requested of the subjects, and are only computed indirectly in the process of evaluating the model likelihoods.

A generative model Bayesian network formalisation of the oddity detection task for the three multisensory observations 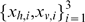 is shown in Fig. 5, where the task is to determine which observation is the odd probe. The graph on the right indicates that the probe visual-haptic observations are related via their common parent, the latent probe stimulus of value 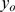. The graph on the left indicates that the four observations composing the other two standard stimuli are all related to the standard stimulus value 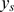. The three different instantiations of this model are given by the different probe hypotheses *o* = 1, 2, 3 which separate the standard and probe stimuli into different clusters. For compactness, we represent this clustering in terms of the *set difference operator* ‘\’. For example, *o* = 3 would mean that stimuli {1,2,3}\3 = {1,2} are drawn from the standard 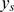, and therefore observations 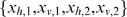 (Fig. 5, left) should be similar to each other – and potentially dissimilar to odd probe observations 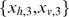 (Fig. 5, right), which were generated independently from 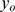. With uniform prior belief about which stimulus o is the odd probe, the ideal Bayesian observer would compute the evidence 
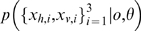
 for each of the three models o as,
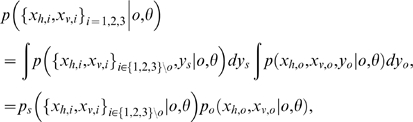
(3)and report the model with the highest likelihood 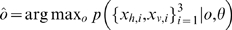. Eq. (3) has two factors 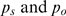, representing the model's explanation of the standard and odd observations respectively after integrating over the unknown true stimuli values 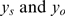. Here, 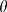 summarises all the fixed model parameters, e.g., the observation variances 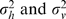. In the event that all distributions involved are Gaussian, eq. (3) is simple to evaluate (see Methods for the detailed parametric form and derivation).

**Figure 6:**
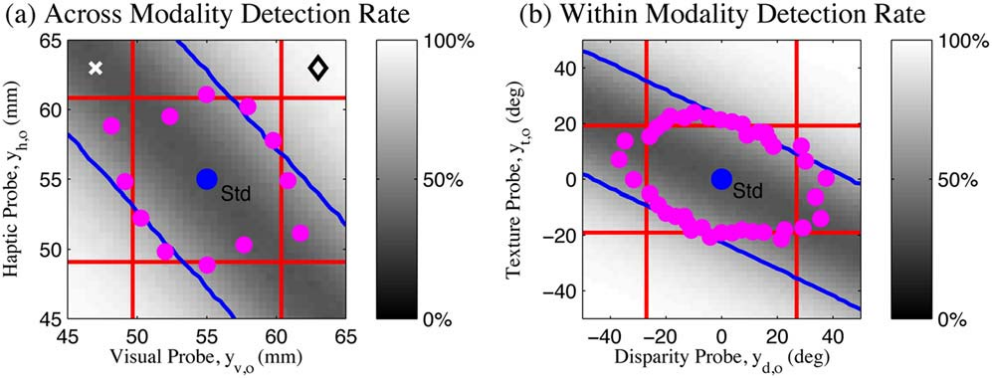
Oddity detection predictions of model selection approach. Oddity detection performance (grey-scale) as a function of probe value for the model selection approach (Fig. 5). Compare the 66% contours (lines) with human performance (dots). Model still predicts an infinite region of non-detection along the cues-discordant diagonal. (a) Across modality visual-haptic experiment. (b) Within modality texture-disparity experiment. Illustrative points correctly (diamond) and incorrectly (cross) classified by model (see text for details).

This model (Fig. 5, eq. (3)) predicts probe detection only outside of the cues-discordant diagonal (Fig. 6a,b, lines), which is still qualitatively similar to the simple factored fusion model (Fig. 3b) and still does not match the data (Fig. 6a,b, points).

Some intuition about how this works can be gained by considering the form of the entire normalised data distribution 
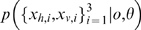
 for each model *o* [20], which in this case factorizes into a standard and probe component (eq. (3)). For example, the model *o* = 3, predicts that the probability mass of the distribution of observations 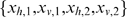 should lie around a four dimensional line through the standard stimuli (where 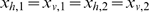) while the distribution of probe observations 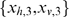 should lie around the line where 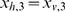 in two-dimensional space. Assuming, for example, that the true model is *o* = 3, then observations at the point indicated by the diamond in Fig. 6a will be correctly classified: The correct model *o* = 3 will have high likelihood as the first four observations will be very similar and lie within the standard probability mass and the two probe observations will be similar to each other and lie within the probe probability mass. An incorrect model, e.g., *o* = 1, will have low likelihood because the observation 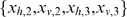 are not at all similar, and so do not lie within the standard probability mass.

Consider instead the point indicated by the cross in Fig. 6a. Here, under the hypothesis that *o* = 3, while the standard observations do lie within the standard probability mass, the discordant probe observations do not lie within the probe probability mass (which was around the line where 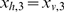), so this hypothesis is unlikely. However, the other hypotheses are also unlikely. For example, consider the alternative *o* = 1, then although 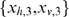 does lie within the probe mass, the remaining observations 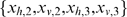 have discordant components and now no longer lie within the standard mass. Therefore no one model is clearly the most probable and detection is unreliable.

### Structure Inference

All of the models discussed so far (Figs. 1 and 5) have assumed a fixed structure. Recent multisensory perception experiments [12], [13], [14], [17], [22], have, however, presented subjects with what is essentially a variable causal structure with respect to the observation correspondence. It is therefore unsurprising that the simple fixed structure ideal observer models have failed to explain the results.

The group of Schirillo, for example, investigated audio-visual spatial localization in humans [12], [13]. Subjects were presented with stimuli from a range of audio and visual stimulus positions; so some were concordant and others were not. They were asked to point out where they thought the audio stimulus came from and whether they thought the visual stimulus co-occurred with the audio stimulus. When the audio and visual stimuli were similar, a unified percept was reported and the reported position was approximately the weighted average of the stimulus as we might expect from maximum likelihood integration [15], [16]. When the stimuli were very discrepant, they were reported to be non-unified, and the position report showed no or negative interaction. The extra uncertainty here is whether the multisensory stimuli did indeed come from the same source or not. This is equivalent to posing uncertain causal structure in the probabilistic model for the ideal observer. We introduced the approach needed to solve this type of problem in multisensory perception as *structure inference* [11]. Kording et al. [15] carried out a detailed analysis of these experiments [12], [13] and showed how the structure inference approach was necessary to explain the results, but termed the procedure *causal inference*.

### Modelling Structure Inference in Oddity Detection

Returning to the oddity experiment of interest, the region of the probe stimulus space not explained by current models is that in which multisensory probe observations are manipulated such that they have implausibly large cross-modal discrepancy. In doing so, they have introduced variability that the models so far (Figs. 1 and 5) cannot represent, so of course they do not predict the data well (Figs. 3 and 6).

The subjects could detect the probe on the discordant-cues axis (on which neither of the models so far can detect the probe) if they can infer this *change in structure* – a potential explanation for the exact source of discrepancy identified earlier between the observed results and our model so far. Indeed in their post experimental analysis, Hillis et al. [17] noted that, “*Sometimes [the subjects] used a difference in perceived size, but frequently they noticed the conflict between the visually and haptically specified sizes and used the perceived conflict to make the oddity discrimination.*” Although unlike [12], [13], Hillis et al. did not systematically ask subjects for their perception of multisensory unity or not for each stimulus, this comment strongly suggests that the subjects in [17] did infer and use the information about the unusual structure in their task (as they have in other related experiments [12], [13], [15]). Next, we formalize how to model the structure uncertainty in oddity detection.

**Figure 7:**
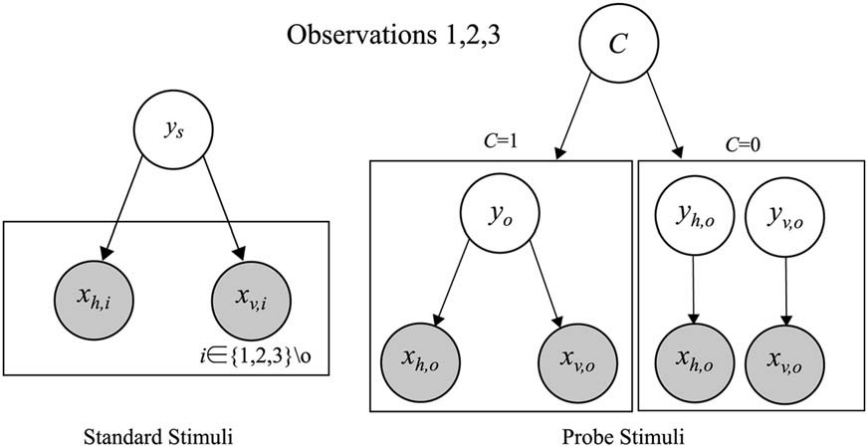
Graphical model for oddity detection via structure inference. Three possible assignments of oddity correspond to three possible models indexed by *o* = 1,2,3. The uncertainty about common causal structure of the probe stimulus is now represented by *C*, which is computed in the process of evaluating the likelihood of each model *o*.

Our model selection interpretation of the oddity detection problem (Fig. 5), can easily be updated to take into account the potential dis-association of the two probe stimulus modalities as shown in Fig. 7. Note that the original simple factored model (Fig. 1) cannot be updated in this way. Here, the Bernoulli association variable *C* has been introduced to represent the uncertain structure: whether the multisensory probe observations have a common source or not. This unavoidably introduces the free parameter 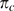 in the prior for *C*, i.e., 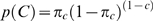. If we were certain a-priori of common causation 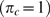, we then have the special case of the model from Fig. 5. If 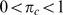, then while computing the evidence for each model 
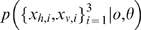
, we integrate over the causal structure *C* (i.e., whether we are feeling and seeing the same thing or not). The exact value of 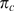 used will depend on the particular combination of senses or cues being used and the particular context and task (and it may vary between people, as do 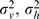 etc). Under the hypotheses of common causal structure *C* = 1, we assume that the two observations 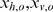 were produced from a single latent variable 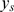, while under the alternate hypothesis *C* = 0, we assume separate sources 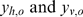 were responsible for each. To evaluate the likelihood of each stimulus being the odd probe *o*, the ideal Bayesian observer would compute and compare the model likelihoods 
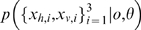
 as follows:
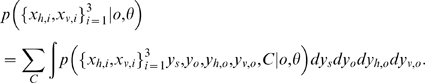
(4)Compared to eq. (3), we now also account for uncertainty in whether we are, for e.g., feeling or seeing the same thing. This is again simple to compute if all the stimulus distributions are Gaussian, requiring only numerical integration of the binary causal structure variable, *C*. The specific parametric solution used is derived in the Methods section.

## Results

To evaluate our multisensory oddity detection model, we compute the success rate distribution produced by our model when detecting the probe, 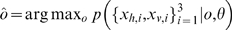, as a function of the probe values 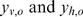. We can then compare the 66% performance thresholds of the model's success rate distribution 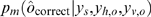 against the human success rate distribution 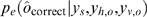 as measured in [17] (Fig. 4, dots). See Methods for details.

### Bayesian Multisensory Oddity Detection Results

#### Detection Threshold Contours

**Figure 8:**
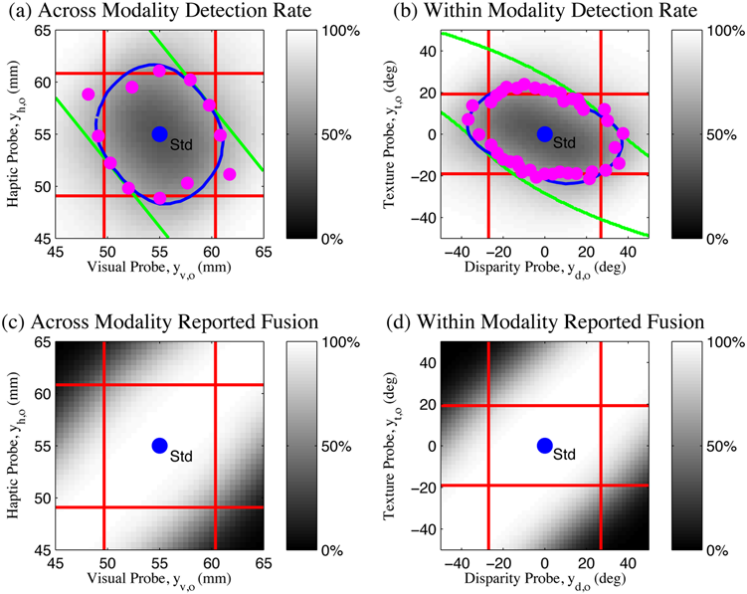
Oddity detection predictions of structure inference approach. (a,b) Oddity detection rate predictions for an ideal Bayesian observer (grey-scale background) using a variable structure model (Fig. 7); Oddity detection contours of the model (blue lines) and human (magenta points) are overlaid with the model prediction from [17] (green lines); Chance = 33%. (c,d) Fusion report rates for ideal observer using variable structure model. Chance = 50%. Across modality conditions are reported in (a,c) and within modality conditions are reported in (b,d).

Figs. 8a and b illustrate the across and within modality results respectively for the two sample subjects from Fig. 4. The experimental data (dots) are shown along with the global performance of the model across the whole input space (grey-scale background, with white indicating 100% success) and the 66% performance contour (blue lines). The human experimental measurements broadly define a region of non-detection centered about the standard stimuli and slanted along the cues discordant line and stretched slightly outside the bounds of the inner uni-modal threshold rectangle. The extent of the non-detection region along this line is increased somewhat in the within modality case as compared to the across modality case [17].

As discussed in the Introduction, none of the simple models – single cue based estimation (Fig. 3a, red lines), mandatory fusion (Fig. 3b, green lines) or combination thereof – explain these particular observations. Moreover, the classical maximum likelihood mandatory fusion theory makes the qualitative error of predicting infinite bands of indiscriminability (Fig. 3, green lines). In contrast, our Bayesian model provides an accurate quantitative fit to the data (Fig. 8, blue lines).

To quantify this, we followed [17] in computing the distance from the standard to each experimental threshold point and the closest predicted threshold along the vector to that point (Fig. 8, points and lines). We could then compare the root mean square error (RMSE) between the experimental threshold distance and the threshold distance predicted by the various models. The qualitative discrepancy between the data and the solely uni-modal or solely mandatory fusion models is clearly highlighted by this measure: Since for many experimental data points there are no predicted thresholds on that vector, these models have infinite error. The two remaining simple models were based on sequentially testing each uni-modal cue independently (Fig. 8a, red rectangle) and sequentially testing the fused estimate followed by each uni-modal cue independently (Fig. 8c, yellow region). We therefore compared our Bayesian model against the sequential uni-modal and sequential fusion models, which had RMSE of 0.8 mm, 0.9 mm and 1.1 mm respectively in the across-modality experiment and RMSE of 2.6deg, 3.9deg and 5.0deg respectively in the within-modality experiment. Our Bayesian ideal observer model therefore provides the best quantitative match to the data as well as the only explanation of the data's specific qualitative form: good performance in quadrants 1&3 as well as a *limited* region of poor performance in quadrants 2&4.

To produce these contours, we coarsely fit the prior probability of fusion 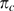 to the data, so as to minimise the contour error, determining 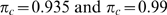 for the across and within modality cases respectively. These values are larger than the 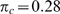 obtained for the related model in [15]. This is understandable, because [15] integrated audio and visual stimuli from distinct locations, which in general should be less correlated than in our case, where stimuli were perceived at the same spatial location. Note also that, as observed, we might expect a stronger prior for fusion within vision, since visual cues at the same retinal location are very likely to be due to the same object, whereas seeing and manipulating different objects simultaneously sometimes occurs.

To gain some intuition into these results, we can again consider the normalised distribution of the data (eq. (4)) under each model here as compared to the fixed structure case discussed in the Introduction, eq. (3). Now, after marginalising over *C*, the probability mass in the probe part of this distribution is a mixture, spread both around 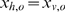 as before (C = 1) and also more uniformly over the space (*C* = 0). Therefore, multisensory observations involving sufficiently discordant points are relatively plausible under the probe distribution, allowing points in quadrant 2&4 to be correctly classified; which was not possible in the example described in the Introduction.

#### Perception of Fusion

To understand clearly how the Bayesian model works, we can also consider its marginal inference for the fusion (common multisensory source) of the probe 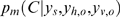, shown in Fig. 8c,d. This corresponds to the human answer to the question “*Do you think your visual and haptic observations are caused by the same object, or have they become discordant?*” This question was unfortunately not asked systematically in [17], but the subjects' self-reporting of a detection of discordant cues is in line with the strategy that falls out of inference with our model.

Along the cues concordant line, the model has sensibly inferred fusion (Fig. 8c,d, quadrants 1&3). In these regions, the model can effectively detect the probe (Fig. 8a,b, quadrants 1&3), and the fused probe estimate 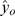 is different to the standard probe estimate 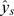.

Considering instead trials moving away from the standard along the cues discordant line, the model eventually infers fission (Fig. 8c,d, quadrants 2&4). The model infers the probe stimuli correctly in these regions (Fig. 8a,b, quadrants 2&4) where the mandatory fusion models cannot (Fig. 8a,b, quadrants 2&4, green lines) because the probe and standard estimates would be the same 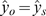. The strength of discrepancy between the cues required before the fission is inferred depends on the variance of the observations 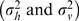 and the strength of the fusion prior 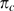, which will vary depending on the particular task and combination of modalities. Data for a total of nine conditions (five across and four within modality) were reported in [17]. The resultant fits of our model to the remaining experiments along with the comparative error analysis (RMSE) to the other models are detailed in the Supporting Information, Text S1 and Fig. S1.

#### Predictions

**Figure 9:**
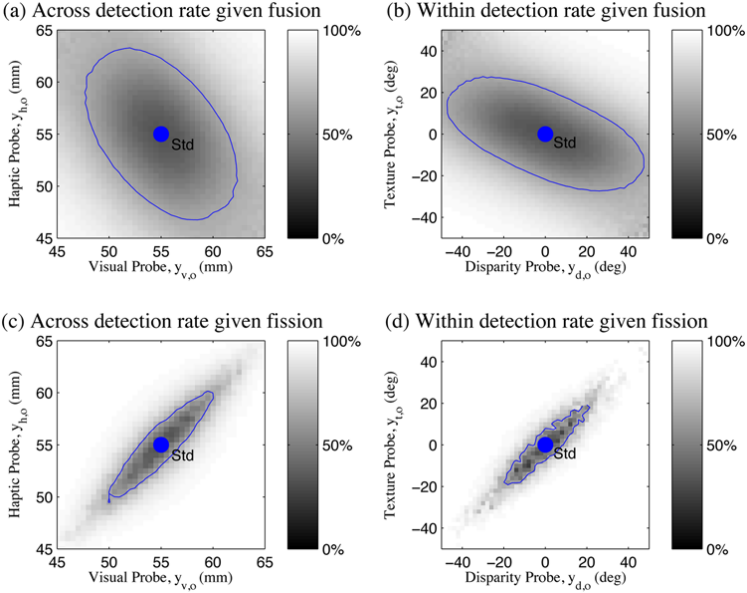
New predictions by the ideal Bayesian observer using the variable structure model. (a,b) Detection rate for trials where fusion was reported (Chance = 33%). (c,d) Detection rate for trials where fission was reported (Chance = 33%). Across-modality condition in (a,c), within modality condition in (b,d). Blue lines indicate contours of detection threshold (66%).

The internal workings of the Bayesian model developed here provide new directly testable predictions about human behaviour in this task. If the participants were also asked for their percept of fusion/fission as well as their oddity estimate (e.g., as in the audio-visual experiments [12], [13]), then the model makes some specific and surprising predictions for oddity detection rate as a function of whether a given trial was also perceived as fused or not. These are illustrated in Fig. 9.

- Although overall performance for detecting probes away from the standard was good (Fig. 8a,b, all quadrants), for those trials where fusion was specifically reported, the discrimination will be more reliable *off* the cues-discordant axis (Fig. 9a,b). Explicitly, see the increased extent of the detection threshold contour along the cues discordant axis in Fig. 9a,b compared to Fig. 8a,b.
- More strikingly, for those trials where fission was reported, the discrimination will only be reliable *off* the cues-concordant axis (Fig. 9c,d). This is the opposite effect to that of trials overall (Fig. 8a,b) and fused trials (Fig. 9a,b). To gain some intuition about this, consider that for a cues-concordant trial to have been inferred as fission, there must have been unusually large noise separating the observations 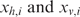 composing the particular multi-modal stimulus *i* which was inferred to be the probe. However, this event would be just as *un*likely to happen to a pair of the true standard observations (causing wrong probe identification) as it would be for the pair of true probe observations. Hence, probe detection under these circumstances would be unreliable.

## Discussion

### Summary

In this paper we have developed a Bayesian ideal observer model for multisensory oddity detection and tested it by re-examining the experiments of Hillis, Ernst, Banks & Landy [17]. In [17], the standard maximum likelihood integration ideal observer approach failed with drastic qualitative discrepancy compared to human performance; however, this was due to simple maximum likelihood fusion being an inappropriate model rather than the failure of ideal observer modelling. The more complete Bayesian ideal observer model developed here provides an accurate quantitative explanation of the data with only one free parameter 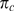, which represents a clearly interpretable quantity: prior probability of common causation. Optimization intuitively sets it to be greater in the within modality case than the across-modality case.

Two novel steps were required to correctly model the multisensory oddity detection problem. The first was the understanding of the problem as a model selection task related to clustering. The unknown bar size or surface slant is of key consequence for the oddity detection, but not directly reported and should therefore be modelled, but integrated over by a Bayesian observer. Our interpretation of the problem is also satisfying in that all the variables in the model represent concrete physical quantities (e.g., haptically observed bar height 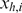 for each object *i*, unknown discrete index *o* of the odd object). This is unlike the analysis in [17] which attempted to model the detection rate contours directly without inference or notion of which particular object *o* was odd: a quantity which the brain is clearly computing since it is the goal of the task. Moreover, within the field of perceptual modelling, we are interested in possible computational mechanisms behind the inference of quantities of interest – in this case ‘oddity’; we have provided an explicit mechanism that may underlie this capability.

The second novel step required was the use of a model with variable structure to appropriately reflect the subject's uncertainty in the causal structure *C* of their observations due to the experimental manipulation. This structure inference approach [11] has recently been used to understand other similarly perplexing experimental results in human audio-visual multisensory perception [12], [13], [14], [15], [22].

In summary, the standard maximum likelihood integration approach to sensor fusion has dramatically failed to explain the experimental data in [17]. This data can now be understood as result of the perceptual system behaving as a Bayesian ideal observer, computing the most likely probabilistic model for noisy data under uncertain causal structure. This theory provides an accurate and intuitive explanation of the data and, via the parameter 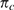, unifies the within and across-modal scenarios.

### Related Research

The framework proposed may seem more complicated than the simple factored cue combination approach (Fig. 1). However, this is necessary and appropriate, because the actual experimental task of oddity detection under causal structure uncertainty is more complicated than the simpler task of stimulus estimation by cue combination. Our approach is parsimonious in that, within the research theme of investigating the extent to which human perception is Bayesian optimal [23], [24], models should use the same generative process as the perceptual experiment. By modelling the three sets of stimuli, including the selection of a probe stimulus and potential disassociation within that stimulus, we have done just this – and provided the best explanation of the data. Finally, despite any apparent complexity, the new model introduces only one new free parameter.

Further studies have investigated stereo-texture fusion [7], [8] for slant perception and visual-haptic fusion [6] for size perception in greater detail, using simpler 2-alternative forced choice paradigms. These have provided further support for the near Bayesian optimality of human multisensory fusion, but only within the domain of small discrepancies where mandatory fusion applies.

Returning to the 3-alternative oddity task, a simple maximum likelihood estimator for uni-modal oddity is the “triangle rule” ([25]). This measures the distances between all three point combinations, discards the two points with minimum distance between them, and nominates the third point as odd. However, this does not provide an acceptable alternative model of the *multisensory* oddity detection scenario studied here as it does not attempt to address the uncertain correspondence between multisensory observations. Specifically, if the multisensory observations were considered to be fused first (eq. (1)), metameric discordant probe observations would still occur – and these cannot be detected by this rule, again producing an infinite band of non-detectability (Fig. 4, green lines). In contrast, if the rule were applied directly to the multisensory observations in two dimensions, there would be no room for fusion effects, and detection would be good throughout, in contrast to the tendency toward fusion illustrated by the human data (Fig. 4, magenta dots).

The theory and practice for modelling uncertain causal structure in inference tasks has a more extensive history in other fields. In artificial intelligence, the theory goes back to Bayesian multinets [26], and is applied today, for example, in building artificial intelligence systems to explicitly understand correlations in multi-party conversations [11]. In radar tracking, this problem is known as data association [27]. Its solutions are used to sort out multiple radar detections, with uncertain causal relation to multiple aeroplanes, into a consistent and accurate estimate of the aircraft locations.

A variety of recent studies have investigated the limits of multisensory cue combination, and have reported “robust” combination, i.e., fusion when the cues are similar and fission when the cues are dissimilar [7], [12]–[14], [22], [28]–[30]. Structure inference models of the type introduced in this paper (and the equivalent models for other experimental paradigms [15]) can in general explain such robust combination results [11]. Some authors have tried to understand robust combination by simply defining a correlated joint prior 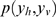 over the multisensory sources like 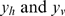. In [29]–[31], this is Gaussian in their difference, reflecting a prior belief that visual and haptic stimuli in the environment are likely to be similar. This prior, however, is insufficient, as it cannot explain complete segregation (complete non-interaction of the observations) observed in many experiments since the jointly Gaussian prior precludes this. Alternately, [28] proposes a joint prior with the special form of a Gaussian-uniform sum to reflect the fact that the observations in the environment are frequently very correlated but sometimes completely unrelated. This is related to our model in that if we chose not to explicitly represent structure *C*, and simplified our generative model as 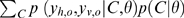, then the joint probability of the visual and haptic stimuli would have qualitatively a Gaussian-uniform sum form. Inference of the probe stimulus values 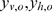 in this case would tend to be fused if the observations 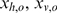 were similar, and be independent if the observations were dissimilar. However, this would be unsatisfactory in our case as the model would be unable to represent all the regimes of the experiment. Moreover, the model would then not explicitly represent the structure *C*, which subjects do infer explicitly as reported in [17] and other related experiments [12], [13]. Another reason for the perceptual system to explicitly represent and infer causal structure is that it may be of intrinsic interest. For example, in an audio-visual context, explicit knowledge of structure corresponds to knowledge of “who said what” in a conversation (for example, see [11]).

A related issue in theoretical modelling of perception is those scenarios in which we expect the prior distribution over an individual stimulus source to be a mixture. For example, Knill [32] considers the case of apparent visual ellipses which may have come from the set of true ellipses or the set of slanted circles. Combined with stereo cues for slant, estimation of ellipse slant also involves non-linear cue combination because of this mixture. However, this is not the same problem as we address in this paper: the correspondence of the multisensory observations or causal model structure in that case is assumed known (Fig. 1), unlike the case studied here (Fig. 7).

One question for future research, which we do not consider here, is that of ancillary cues and their impact on model parameters. Ancillary cues are frequently considered in their role of providing information about the reliability of the main cues for weighted averaging [2]. They could also affect the parameters of the structure inference procedure. As an example, the strength of the fusion prior 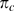 might decrease with the spatial discrepancy of the visual and haptic cues [33].

How might the perceptual system's neural architecture perform the computations proposed in this paper to solve the oddity detection problem? Work on probabilistic population coding describes how neural populations could represent and compute with probability distributions such as those used here [34], [35]. For the computations involved in multisensory integration, we need to compute products of probability distributions; indeed, population codes represent-able by neurons with Poisson firing statistics would be particularly well suited for rapid computation of such operations [36]. Further experimental work is needed to confirm whether any of these proposed population coding models are actually implemented by biological neural networks.

### Conclusions

In this paper, we have derived a Bayesian model for multisensory oddity detection which exploits structure inference [11], [15], [16]. With this model, we are able to understand the results of experiments on human multisensory oddity detection [17] which the classical maximum likelihood integration theory, and other simpler theories for cue combination, fails to model with drastic qualitative discrepancy. Moreover, the structure inference approach unifies the existing discrepant results for across and within-modality scenarios – and makes new testable predictions for further experiments.

In addition to the audio-visual domain and direct estimation paradigm investigated by related work [15], [16], we have now provided evidence that structure inference occurs in combining visual-haptic as well as texture-disparity observations, and does so in a completely different oddity detection paradigm. The commonality of this collection of results – across and within different types of modalities, and across different experimental paradigms – begins to suggest that structure inference may actually be a commonly evolved principle for combining perceptual information in the brain.

## Methods

### Setting Model Parameters

Our model contained four parameters: The noise level of each modality (for e.g., 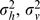), the prior belief about the distribution of bar heights (*y*), and the prior probability of fusion 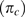. The standard approach for sensory integration modelling (e.g., refer [2], [5]) is to determine the variances in each modality independently in uni-modal experiments, thereby eliminating them as free parameters. In our case, this involves simulating the uni-modal experiments and matching the outcome to the uni-modal experimental results (Fig. 4, red lines). Specifically, we take the model of eq. (4), Fig. 7 and consider only one modality at a time (without using the extra structure variable as this is only relevant for multi-modal observations). For any given setting of 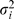, we can simulate the whole uni-modal experiment and measure the 66% performance threshold. So, we simply perform a one dimensional search to find the value of 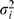 which produces the threshold most closely matching the uni-modal experimental data (Fig. 4, red lines).

For a Bayesian model, we are unavoidably required to specify some prior belief about the latent stimulus sizes *y*, and it is mathematically convenient for these to also use a Gaussian parametric form 
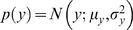
. We use the same distribution for all the latent *y*. We assume subjects have correctly estimated the true mean 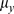 of the latent distributions, which is the standard stimulus: 55 mm in the intra-modal experiment and 0 deg in the inter-modal experiment. The variance 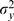 of the subjects' prior belief is slightly harder to determine. We use an uninformative prior for all subjects for each experiment 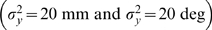 to ensure that the whole state space investigated by the experiment was plausible under the prior distribution. Subsequent detailed analysis showed that, unlike for 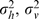, the results are highly insensitive to the specific value of 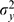.

Finally, we expect the prior probability of fusion 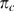 to be dependent on the individual subject and the modality pair in question. We coarsely fit 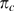 for each subject and experimental condition to minimise the mean square error between the predicted and experimental contours.

### Simulating Perceptual Noise

Human subjects' decisions in this task are noisy because they are estimating oddity based on the noisy perceived samples 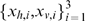 of the experimentally controlled stimuli 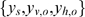. To correctly model this task, it is therefore insufficient to simply control 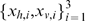 and compute the model's response 
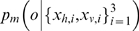
, since it is the human's response to the experimentally controlled stimuli 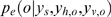 that is reported in experiments. To produce comparable results for the model 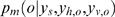, we simulate the noisy perceptual process as well as oddity estimation, integrating over the actual noisy observations 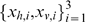 as follows:
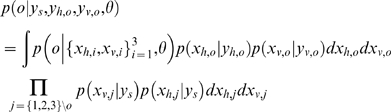
We approximate this by sampling 50,000 noisy observations 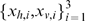 for every probe condition 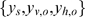 and averaging over the response of the model to each sample. The importance of correctly simulating the noise processes in psychophysics models was recently discussed in the analysis of a related experiment [15]. The measured 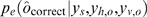 for human subjects can now be correctly and directly compared to the success rate of the model 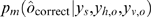.

### Optimal Oddity Inference with Variable Structure Derivation

We assume all the observations are distributed normally given the source 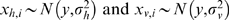, and that the subject's prior belief about the source locations is represented by 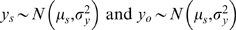. Conditioned on the causal structure 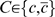 as well as the model (oddity) 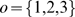, the likelihood of oddity factors into standard 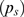 and odd 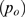 components, each of which is determined by an integral of Gaussian products. Each component represents the ultimate likelihood of each observation 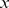 given the noisy perceptual process 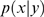 and prior uncertainty about the stimulus 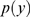. Writing for brevity in terms of precisions 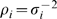 rather than variances 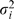, and assuming that 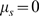, the model likelihood 
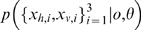
 can be written as follows:
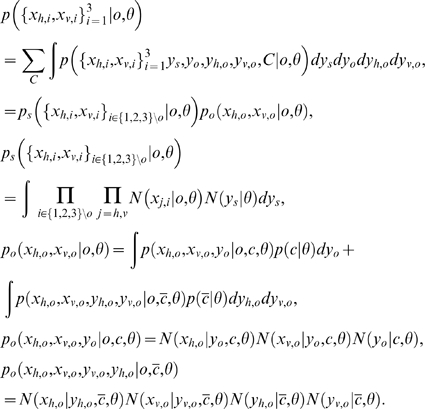
To illustrate a concrete example, to compute the likelihood of hypothesis that the stimuli number three is odd, the three required terms are:
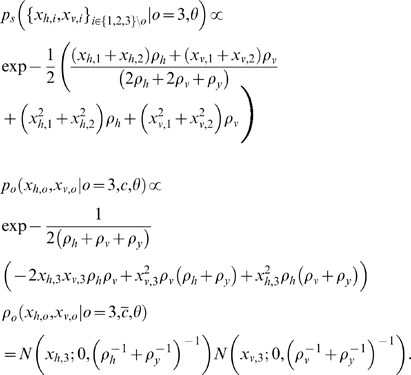
For the special case of known correspondence considered in Eq. (3) and Fig. 5, the above equations are simply conditioned on 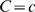, i.e., 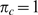.

## Supporting Information

Text S1 Supporting Information for “Multisensory Oddity Detection as Bayesian Inference”
(0.06 MB DOC)

Figure S1 Complete oddity detection predictions of structure inference approach. Oddity detection rate threshold contours for the Bayesian model (blue lines), mandatory fusion model (green lines) and uni-modal model (red lines) are shown along with human thresholds (magenta points). (a–d) Visual-haptic condition. (e–h) Texture-disparity condition. Chance = 33%. Contour root mean squared error is given for; E_b_ : Bayesian model, E_mf_ : sequential fused estimate and uni-modal model, E_um_ : sequential uni-modal model.
(2.18 MB TIF)
