# Supplementary material for: Multisensory Oddity Detection as Bayesian Inference
Source: PLoS One. 2009 Jan 15;4(1):e4205. doi: 10.1371/journal.pone.0004205 (PMC2625446; doi:10.1371/journal.pone.0004205)
Supplement: Text S1 — Supporting Information for “Multisensory Oddity Detection as Bayesian Inference” (0.06 MB DOC) [file pone.0004205.s001.doc]

# Multisensory Oddity Detection as Bayesian Inference

## Supporting Information

### Complete Results

In this section, we summarize the predictions of our model and the experimental data for all the remaining scenarios of the nine reported in [17]. In Fig. S1a-d, the across-modality predictions are shown for the four different subjects tested in addition to the first subject shown in Fig. 4a. The uni-modal variances (determined by the red lines) and prior probability of fusion ($\pi_{c}$={0.845, 0.998, 0.85, 0.94} – computed from data fits) vary by subject, and enable accurate prediction of the multi-modal detection contours in almost every case. In Fig. S1e-h, the within modality predictions are shown for two subjects (different to those from the across-modality experiment) tested under various viewing conditions (of distance and base slant, see [17] for details). In these cases, $\pi_{c}$={0.99, 0.94, 0.9995, 0.9999} provided the best fit to the data.

### Changing cue variance

Our approach required one compromise in modelling power compared to other studies of texture cues for slant. These cues exhibit the unusual property of being perceived with less variance as a function of the actual stimulus slant. So the observations should ideally be modelled as $x_{t}\sim N(y_{t},\sigma_{t}(y_{t}))$where $\sigma_{t}(y_{t})=\alpha\exp(-\beta\left|y_{t}\right|)$is a decreasing function of the absolute magnitude of$y_{t}$. Other studies have investigated and modeled this phenomenon in detail [37,7,8]. This is the reason why human data in the within-modal experiments (Fig. S1e-h) takes a more curved shape than the across-modal experiments (Fig. S1a-d). This is particularly apparent when the standard stimulus is taken not to be at (0,0) since, in those cases, the variances of observations above and below the standard are not symmetrical (Fig. S1f-h).

The simpler model in [17] allowed incorporating of the dependence of $\sigma_{t}(y_{t})$ on $y_{t}$ numerically, while still retaining computational tractability (hence, the curved green prediction lines in Fig. 4b). Note, however, that this introduces additional free parameters in the function$\sigma_{t}(y_{t})$, which are fit to the data. After taking this into account, our model (Fig. 8, blue lines) has fewer free parameters than the model used in [17] (Fig. 8, green lines) despite providing a qualitatively and quantitatively better fit to the data.

In our approach, we were not able to incorporate variable and asymmetric variance while retaining analytical and computational tractability of the model, and we simply assumed it was constant and symmetric. Hence, the fits of our model to the within-modal data with asymmetric variance (Fig. S1f-h) do not have the same quantitative accuracy as for the other experiments (Fig. S1a-e). Nevertheless, even in these cases, the essential slightly elongated region of non-detection along the cues-discordant axis is still captured albeit without the curve related to$\sigma_{t}(y_{t})$. In future work, this limitation could be potentially addressed while retaining the same general framework by including a parameterised dependency $\sigma_{t}(y_{t})$ in the generative model and applying a sampling, rather than analytical, approach to integrating the latent variables $\{y_{o},y_{t,o},y_{d,o},y_{s}\}$ in Eq. (4).

### Figure Legends

Figure S1: **Complete oddity detection predictions of structure inference approach.** Oddity detection rate threshold contours for the Bayesian model (blue lines). Mandatory fusion model (green lines) and uni-modal model (red lines) are shown along with human thresholds (magenta points). (a-d) Visual-haptic condition. (e-h) Texture-disparity condition. Chance=33%. Contour root mean squared error is given for Bayesian model ($E_{b}$), sequential fused estimate and uni-modal model ($E_{mf}$) and sequential uni-modal model ($E_{um}$).
