# Supplementary figures and images for: Multisensory Oddity Detection as Bayesian Inference
Source: PLoS One. 2009 Jan 15;4(1):e4205. doi: 10.1371/journal.pone.0004205 (PMC2625446; doi:10.1371/journal.pone.0004205)

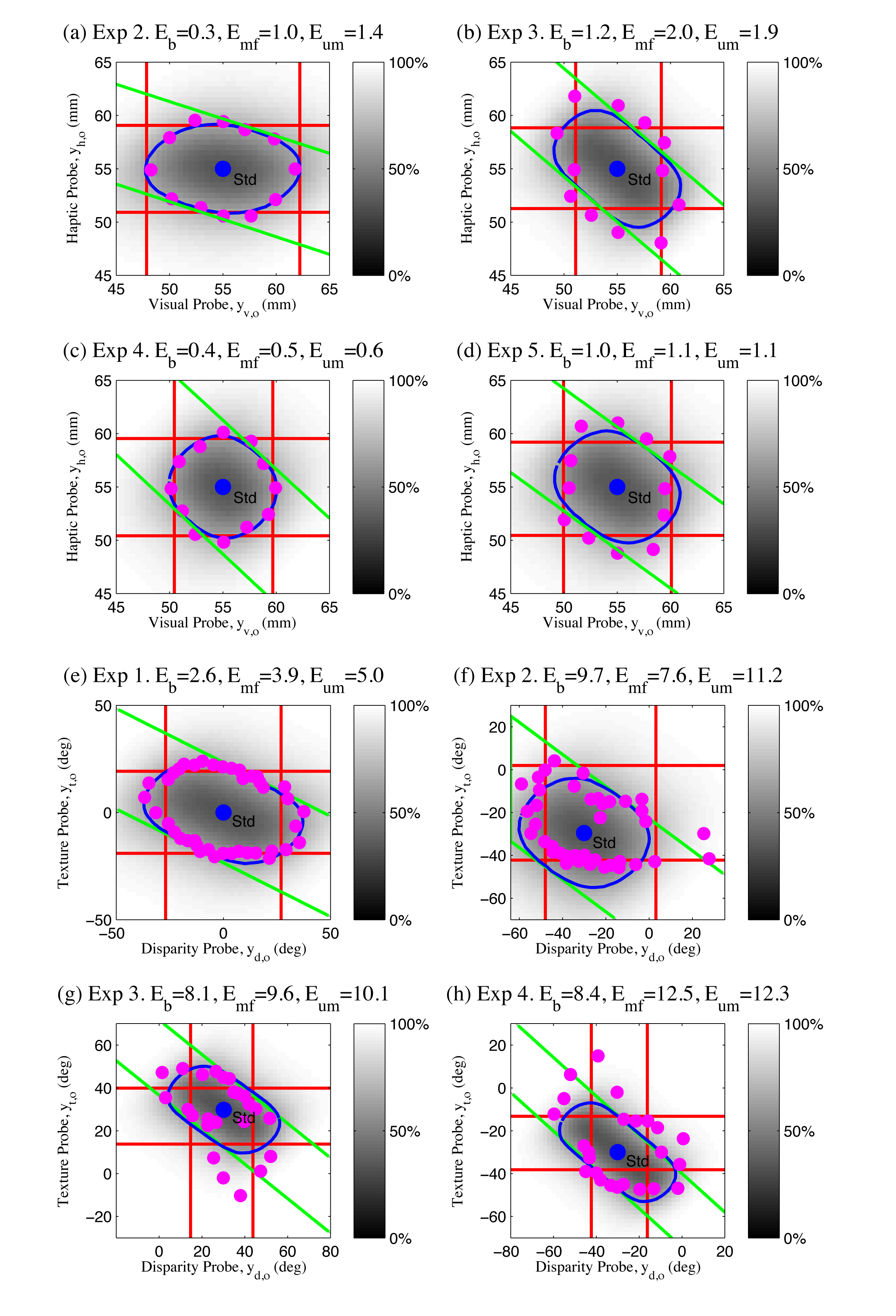

Supplement: Figure S1 — Complete oddity detection predictions of structure inference approach. Oddity detection rate threshold contours for the Bayesian model (blue lines), mandatory fusion model (green lines) and uni-modal model (red lines) are shown along with human thresholds (magenta points). (a–d) Visual-haptic condition. (e–h) Texture-disparity condition. Chance = 33%. Contour root mean squared error is given for; Eb : Bayesian model, Emf : sequential fused estimate and uni-modal model, Eum : sequential uni-modal model. (2.18 MB TIF) [file pone.0004205.s002.tif]
